# Intra- and interobserver concordance of a new classification system for myopic maculopathy

**DOI:** 10.1186/s12886-021-01940-4

**Published:** 2021-04-23

**Authors:** Rong-rong Zhang, Yan Yu, Yin-fen Hou, Chang-fan Wu

**Affiliations:** Department of Ophthalmology, Yijishan Hospital of Wannan Medical College, 92 West Zheshan Road, Wuhu, 241001 Anhui Province People’s Republic of China

**Keywords:** High myopia, Myopic maculopathy, ATN classification, Optical coherence tomography, Agreement study

## Abstract

**Background:**

Myopic maculopathy (MM) is one of the major causes of visual impairment and irreversible blindness in eyes with pathologic myopia (PM). However, the classification of each type of lesion associated with MM has not been determined. Recently, a new MM classification system, known as the ATN grading and classification system, was proposed; it is based on the fundus photographs and optical coherence tomography (OCT) images and includes three variable components: atrophy (A), traction (T), and neovascularization (N). This study aimed to perform an independent evaluation of interobserver and intraobserver agreement for the recently developed ATN grading system for MM.

**Methods:**

This was a retrospective study. Fundus photographs and OCT images of 125 patients (226 eyes) with various MMs were evaluated and classified using the ATN grading of the new MM classification system by four blinded and independent evaluators (2 attending ophthalmologists and 2 ophthalmic residents). All cases were randomly re-evaluated by the same observers after an interval of 6 weeks. The kappa coefficient (κ) and 95% confidence interval (CI) were used to determine the interobserver and intraobserver agreement.

**Results:**

The interobserver agreement was substantial when considering the maculopathy type (A, T, and N). The weighted Fleiss κ values for each MM type (A, T, and N) were 0.651 (95% CI: 0.602–0.700), 0.734 (95% CI: 0.689–0.779), and 0.702 (95% CI: 0.649–0.755), respectively. The interobserver agreement when considering the subtypes was good or excellent, except for stages A1, A2, and N1, in which the weighted κ value was less than 0.6, with moderate agreement. The intraobserver agreement of types and subtypes was excellent, with κ > 0.8. No significant differences were observed between the attending ophthalmologists and residents for interobserver reliability or intraobserver reproducibility.

**Conclusions:**

The ATN classification allows an adequate agreement among ophthalmologists with different qualifications and by the same observer on separate occasions. Future prospective studies should further evaluate whether this classification can be better implemented in clinical decision-making and disease progression assessments.

**Supplementary Information:**

The online version contains supplementary material available at 10.1186/s12886-021-01940-4.

## Background

Myopia has been one of the global public health problems leading to visual impairment and blinding complications, especially in East Asia [1]. By 2050, approximately 4.76 billion people (49.8%) are expected to have myopia, and up to 938 million people (19.7%) will have high myopia (HM) [2]. The prevalence of PM has been reported to be 0.9–3.1%, and PM is a leading cause of irreversible visual impairment in East Asia [3–5].

Myopic maculopathy (MM) is one of the major causes of visual impairment and irreversible blindness in eyes with PM and is predicted to impact approximately 55.7 million people and up to 18.5 million people worldwide in 2050, respectively [6, 7]. Although MM is clinically important, the classification of each type of lesion associated with MM has not been determined, and the current classification systems for MM cannot entirely explain the various changes that occur in the patient’s macula. The META-PM classification of MM established by the International Pathologic Myopia Study Group in 2015 is currently still accepted worldwide; it is just based on color fundus photography [8], and other myopic macular lesions, such as myopic traction maculopathy (MTM) and dome shaped macula (DSM) were also not included in this classification system [9, 10]. In addition, fundus images may look different due to the different background pigmentations of ethnic groups and the use of different examinations, which could affect a reliable diagnosis of fundus lesions.

With the development of fundus examinations, the availability of new OCT equipment facilitates better detection of subtle changes and early signs of MM, which is not only helpful for the definition and classification of types but also helpful for the evaluation of natural processes [11]. Recently, Parolini et al. [12] retrospectively evaluated 281 eyes with MTM over 11 years and proposed a comprehensive OCT-based classification of MTM, namely, the Myopic Traction Maculopathy Staging System (MSS). The MSS system includes four MTM retinal stages and three foveal stages, focuses on the evolving dynamic nature of the disease and proposes a practical guide for the treatment of MTM [13]. Different from the previous choice on the various surgical methods that have been reported in the treatment of MTM [14–17], therapy based on the MSS system could offer the greatest anatomical and functional improvement, involving selection among pars plana vitrectomy (PPV), macular buckle (MB) or combined MB and PPV for efficient treatment per each stage of MTM [18]. However, MTM represents only traction lesion, which is just one type of fundus damage in MM, and does not fully cover all the pathological changes of MM.

Considering the shortcomings of the META-PM system and the convenience of the OCT technology, a new MM classification system was proposed, known as the ATN grading and classification system, which includes three variable components: atrophy (A), traction (T), and neovascularization (N) [19]. This new grading system has an efficient and comprehensive approach that relates fundus photographs and OCT images to a more precise definition of disease stages and grading management and further provides great value for the early prevention of disease, selection of surgical methods and evaluation of prognosis. Due to the ATN classification being published recently, no additional clinical application or observation studies have been conducted. Many studies have analysed the risk factors and progressive pattern of MM based on META-PM [20–22], and no research has been conducted within the same HM population to comprehensively analyse the development pattern of the MM based on the new MM grading system. Agreement analysis was just performed by retinal specialists to validate the ATN classification system [23]. Therefore, the aim of the present study was to perform an independent inter- and intraobserver agreement analyses to validate the ATN grading and classification system in two different levels of evaluators, to provide more reliable evidence for the clinical application of the ATN classification and to enable the comprehensive classification system of ATN to be more widely used in the diagnosis and treatment of MM.

## Methods

### Study population

This retrospective study collected 125 patients (226 eyes) with HM who underwent fundus photographs and OCT examinations at our hospital, including 62 males and 63 females. To perform an adequate agreement study, patients with all types of retinal and choroidal lesions as defined by the recently proposed ATN classification system were included. The inclusion criteria included refractive error ≤ − 6.0 D or axial length ≥ 26.0 mm with an atrophy degree greater than or equal to grade 1 on the three components (atrophy, traction, or neovascularization) of the ATN classification. The exclusion criteria were other retinal or choroidal disorders, such as diabetic retinopathy; retinal vascular diseases, including retinal vein occlusions and age-related macular degeneration; poor quality of fundus and OCT images; and a history of vitreoretinal surgery.

### Ophthalmic examinations

Comprehensive ophthalmologic examinations were performed in all participants. An autorefractometer (Topcon Corp, Tokyo, Japan) was used for spherical equivalent refraction measurements, and axial length was recorded using the IOLMaster (Carl Zeiss Meditec, Jena, Germany). Dilated 45°digital color fundus photographs (centred on the macula) were taken using a TRC-50DX (Topcon Corp, Tokyo, Japan). Vertical and horizontal scans that passed through the centre of the fovea and raster scans covering all the macular complications were acquired using the spectral domain OCT (Heidelberg Engineering, Heidelberg, Germany).

### ATN classification

This classification system was based on the fundus photographs and OCT images, classified the MM into three major lesions: atrophy (A), traction (T) and neovascularization (N). Atrophy components include: A0-no myopic retinal lesions; A1-tessellated fundus only, refers to the visibility of large choroidal vessels at the posterior fundus pole outside of the peripapillary region; A2-diffuse chorioretinal atrophy, refers to the yellowish-white and ill-defined appearance of the posterior pole; A3-patchy chorioretinal atrophy, refers to a grayish-white and well-defined atrophy lesion in the macular area or around the optic disc; A4-complete macular atrophy, refers to a well-defined, round chorioretinal atrophic lesion which is grayish-white or whitish and appears around a regressed fibrovascular membrane. Traction components include: T0-no macular schisis; T1-inner or outer foveoschisis, refers to a thickening of the inner retinal or outer retinal layers in a column-like structure, at different levels, from the inner nuclear layer to the internal limiting membrane (ILM) or the outer nuclear layer to the external limiting membrane (ELM), respectively; T2-inner and outer foveoschisis; T3-foveal detachment, refers to the upper edge of the external retina was further elevated and attached to the upper part of the retinoschisis layer by further enlargement of the detachment; T4-full-thickness macular hole, refers to an anatomic defect in the fovea featuring interruption of all neuralretinal layers from the ILM to the retinal pigment epithelium (RPE); T5-macular hole + retinal detachment, refers to a neurosensory detachment of the macula with separation of the photoreceptors from the RPE due to macular hole. Neovascularization components include: N0- no myopic choroidal neovascularization (CNV); N1-macular lacquer cracks, refers to the irregular, yellowish linear lesions often branching and crisscrossing in the macula; N2a-active CNV, refers to a flat, small, greyish subretinal lesion beneath or in close proximity to the fovea with or without haemorrhage; N2b-scar or Fuch’s spot, refers to a grayish-white scars without associated exudation and sometimes with associated pigmentation.

### Evaluation procedures

The assessment was performed by four ophthalmologists representing two different levels of experience in MM: two senior fellowship-trained ophthalmologists and two ophthalmic residents. Each evaluator was trained in this new classification system (S.figure) before performing the assessment, and they were provided a description and pictorial representation of the ATN classification from the original article as a reference to be used when performing each reading [19]. All fundus photographs and OCT images of each patient were blinded and provided to the observer in random order. The four observers independently evaluated all images and were also blinded to the reading of the other evaluators, as well as to patient information. No time limitations were implemented on any of the readings, but the first round of evaluation were completed within 1 month at the latest. Six weeks after the completion of the first-round evaluation, without pretest training, the same images were presented in a different sequence and re-evaluated following the same procedures. This study adhered to the principles of the Declaration of Helsinki, and the retrospective review of patient records was approved by the Ethics Committee of Wannan Medical College Yijishan Hospital (Approval No.2019-052).

### Statistical analysis

Statistical analysis was conducted using SPSS for Windows version 22.0 (SPSS, Inc., Chicago, IL). The κ coefficient is the most commonly used agreement statistic in medical studies, as it represents the magnitude of exact agreement between different evaluators with correction by chance. Interobserver agreement was achieved by comparing the initial responses of all evaluators. Intraobserver agreement was determined by comparing the responses of the same evaluator for two assessments of the same cases, which were presented in a random sequence after an interval of 6 weeks. The kappa coefficient (κ) was used to identify inter- and intraobserver agreement. The agreement was initially evaluated at the main-type level (A, T and N) and then at the subtype level for A, T and N lesions. Levels of agreement for κ were described by Landis and Koch [24], with κ values 0.00–0.20 considered slight agreement; 0.21–0.40, fair agreement; 0.41–0.60, moderate agreement; 0.61–0.80, substantial agreement; and 0.81–1.00, almost excellent agreement. The κ values are presented with 95% confidence intervals (CIs).

## Results

### Demographic and clinical characteristics

A total of 125 consecutive patients with HM were collected, and the fundus photographs and OCT images of 226 eyes were assessed and graded by two different levels of evaluators. For all eyes, any of the three components (A, T and N) could be stage 0 or higher except component A (stage ≥1). The average age of the patients was 56.53 ± 16.76 years, with a range of 18 to 94 years. The mean spherical equivalent was − 12.6 ± 4.5 D, with a range of − 6.0 to − 24.0 D, and the mean axial length was 29.4 ± 3.08 mm, with a range of 26.0 to 35.2 mm.

### Interobserver reliability

The total interobserver agreement is shown in Table 1, and the weighted Fleiss κ values for each MM type (A, T, and N) were 0.651 (95% CI: 0.602–0.700), 0.734 (95% CI: 0.689–0.779), and 0.702 (95% CI: 0.649–0.755), respectively. These values were considered substantial agreement for type A, T and N lesions. Although there were no significant differences between attending surgeons and residents in the specific grading agreement of A, T, and N type lesions, the weighted κ values of the residents were lower than those of the attending surgeons (Table 2). Agreement for each subtype was good or excellent, ranging from κ =0.662 to κ = 0.835, except for stages A1, A2, and N1, for which the weighted κ value was less than 0.6, with a moderate agreement (Table 3).

**Table 1:** Inter-observer agreement for each lesion type

Types	Weighted Fleiss κ	95% CI
A	0.651	0.602–0.700
T	0.734	0.689–0.779
N	0.702	0.649–0.755

**Table 2:** Inter-observer and intra-observer agreement (κ) according to the level of training

	Inter-observer	Intra-observer
	κ (95%CI)	κ (95%CI)
Attendings		
A	0.764 (0.670–0.858)	0.824 (0.765–0.883)
T	0.836 (0.771–0.901)	0.866 (0.819–0.913)
N	0.819 (0.727–0.911)	0.892 (0.829–0.955)
Residents		
A	0.594 (0.482–0.706)	0.796 (0.698–0.894)
T	0.715 (0.599–0.831)	0.853 (0.812–0.894)
N	0.624 (0.538–0.710)	0.851 (0.796–0.906)

**Table 3:** Inter-observer and intra-observer agreement for each lesion sub-type

Sub-types	Inter-observer		Intra-observer	
	κ	95%CI	κ	95%CI
A1	0.563	0.465–0.661	0.885	0.834–0.936
A2	0.529	0.402–0.656	0.889	0.840–0.938
A3	0.747	0.616–0.878	0.904	0.845–0.963
A4	0.722	0.599–0.845	0.896	0.843–0.949
T0	0.729	0.609–0.849	0.960	0.931–0.989
T1	0.713	0.566–0.860	0.892	0.794–0.990
T2	0.711	0.595–0.827	0.893	0.820–0.966
T3	0.662	0.568–0.756	0.873	0.785–0.961
T4	0.762	0.615–0.909	0.890	0.812–0.968
T5	0.829	0.666–0.992	0.903	0.819–0.987
N0	0.742	0.560–0.924	0.917	0.858–0.976
N1	0.471	0.393–0.549	0.884	0.826–0.943
N2a	0.763	0.630–0.896	0.901	0.855–0.948
N2s	0.835	0.706–0.964	0.893	0.820–0.966

### Intraobserver reproducibility

The intraobserver agreement and weighted κ values for the 226 images are shown in Tables 2 and 3. In the repeated evaluation 6 weeks after the first assessment, we did not observe significant differences in the intraobserver agreement of specific A, T and N type lesions between the attending surgeons and residents. When we evaluated the level of agreement according to the subtype level, the intraobserver agreement was excellent (κ > 0.8). The detailed intraobserver agreement by subtype level is shown in Table 3.

### Illustrate with examples

**Fig. 1:**
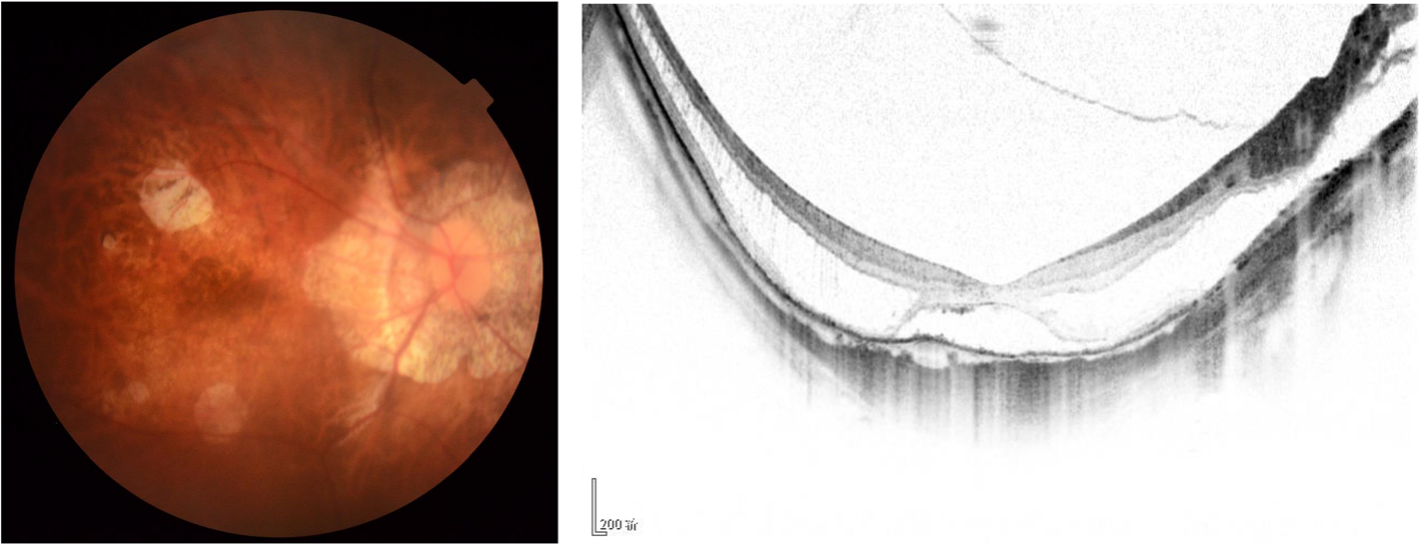
Highly myopic eye with patchy atrophy, foveal detachment, and no signs of choroidal neovascularization (CNV) would be classifndied as stage A3T3N0 both in ophthalmologists with different qualifications

**Fig. 2:**
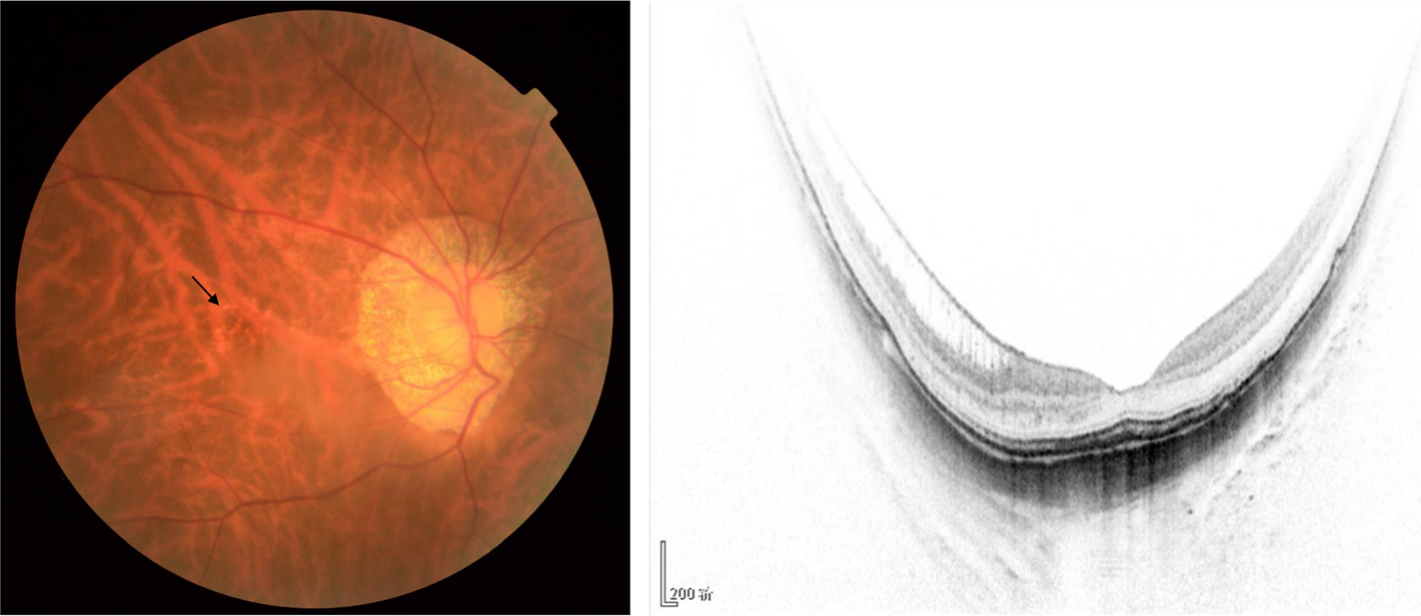
Highly myopic eye with tessellated fundus, inner foveoschisis, and macular lacquer cracks (black arrow) would be classified as stage A1T1N1 by attending ophthalmologists, while classified as stage A1T1N0 by ophthalmic residents

Figures 1 and 2 present an example of the classification of two different eyes. Figure 1 depicts an eye with patchy atrophy, foveal detachment, and no signs of choroidal neovascularization (CNV), which was classified as stage A3T3N0 by all four ophthalmologists. Figure 2 shows a highly myopic eye with tessellated fundus, inner foveoschisis, and macular lacquer cracks (black arrow), which was classified as stage A1T1N1 by the attending ophthalmologists and as stage A1T1N0 by the ophthalmic residents.

## Discussion

In this study, we performed an independent inter- and intraobserver agreement assessment of the ATN grading and classification system for MM. The full interobserver agreement in our study was substantial for each MM type (A, T, and N), and the intraobserver agreement was excellent. In addition, half of our evaluators were residents, and even though their agreement was not significantly different from that of the attending ophthalmologists, the ophthalmic residents exhibited κ values considered indicative of moderate or substantial agreement less than the attending ophthalmologists, who had higher κ values considered indicative of substantial or excellent agreement. These results upheld the validity and reproducibility of the recently proposed ATN classification system for MM.

In our study, the results showed that the level of interobserver agreement was slightly lower in the atrophic component than in the other components among the attending ophthalmologists and ophthalmic residents, which might have been influenced by the A1 and A2 grading. This finding was consistent with the agreement reported by the authors who developed this classification [23], but the level of interobserver agreement in our study was just slightly lower than that in their study (A: κ =0.753; T: κ =0.847; N: κ =0.849). A possible reason for this outcome could be the small sample (60 eyes) analysed and retinal specialist involvement. When analysing the interobserver agreement at the subtype level of each type, we could be found that the κ value at stages A1, A2 and N1 was considered to indicate moderate agreement, while other subtype were substantial or excellent. This outcome was similar to the recently reported data from the study by Ruiz-Medrano et al. [23], which verified that the disease migration from stage A1 to stage A2 was slow, and differentiating these two categories via color fundus photographs only was difficult. Therefore, they proposed that combining these two stages (A1 and A2) into a single stage in the ATN classification system was better. However, early research on the long-term development pattern of MM have found that the initial sign of highly myopic eye progression to the MM stage was the occurrence of a tessellated fundus that could develop diffuse atrophy, lacquer cracks, or more typically CNV formation over time [25]. In addition, the clinical characteristics of these two categories were also different; patients with a tessellated fundus were young and had significantly better visual acuity, while patients who developed diffuse chorioretinal atrophy were generally over 40 years old and had poor vision [20]. Therefore, we believe that it is reasonable for the ATN system to classify the tessellated fundus and diffuse chorioretinal atrophy into two grades, considering the pathological features and progression trends of these two categories based on the earlier studies. At present, the key to discriminating between these two categories is to identify relatively objective quantitative indicators rather than to only examine color changes in fundus photographs. Fang et al. [26] reported that the choroidal thickness (CT) was dramatically diminished from the tessellation to peripapillary diffuse choroidal atrophy (PDCA) only in the nasal location, and the cut-off value of CT to evaluate eyes with PDCA from tessellation was 56.5 μm nasal to the fovea, which was useful in differentiating these two categories. Therefore, the cut-off CT value can be used as a supplement to fundus photography for accurate diagnosis of a tessellated fundus and diffuse chorioretinal atrophy lesions.

The present study also found that the interobserver agreement for the N1 level was moderate, and we speculate that a possible reason might be due to the absence of uniform diagnostic criteria and the different observation indices of the two diagnostic tools. Lacquer cracks (LCs) in the fundus showed irregular, yellowish linear lesions often branching and crisscrossing in the macula, while OCT showed discontinuities of retinal pigment epithelium [27]. The evaluator used different types of images and standard grading N1, which led to some deviation in the results. In addition, previous studies have claimed that LCs are risk factors for CNV [21, 25, 28]; however, the progression of LCs into CNV does not appear common (13%), and most LCs develop patchy atrophy (43%) [25]. Research on posterior fundus changes in PM also found that LCs often appeared at the early stage of MM, and the young individuals often do not have noticeable staphyloma or early atrophic changes of the retina [29]. However, the ATN system classified LCs into the neovascular group, which may lead to the classification of some LCs that may develop into patchy atrophy as a neovascular lesion and further impact the validity of this classification system in a long-term progress observation [30]. Consequently, further study to determine the standard of LC identification and modify the LC classification is necessary and important.

The main limitations of this study are the limited number of samples at certain stages (such as stages T3, T4, and N1) and the limited OCT images (just centred on the macula), which relatively affected the study results. In some cases, the appearance of the three components in fundus photographs and OCT images may have an interactive effect. When macular hemorrhage occurs in the fundus, the T lesion may be affected and frequently misdiagnosed as foveal detachment (T3), and macular holes with retinal detachment (T5) lesions often affect the fundus photograph quality, which leads to grade A stage being difficult to identify. Chen and colleagues [30], in their research on the morphological characteristics of and risk factors for MM, proposed that the coexistence of the three components could influence the correlation between risk factors and specific types of MM. Therefore, determining the impact of coexistence of the three components, whether for accurate diagnosis or observation of the progression of MM, is still a challenge. In contrast, the strengths of this study design included substantially large sample sizes and the participation of observers with different qualifications.

## Conclusion

In conclusion, this study validates the reliability of the recently proposed ATN classification system, with relatively high interobserver and intraobserver agreement among ophthalmologists with different qualifications. The ATN classification system includes three main components (atrophy, traction, and neovascularization) of the fundus lesions, enabling comprehensive and efficient comparisons of findings from clinical trials and epidemiologic studies, improving the diagnosis and grading management of MM, and providing greater value for research on MM progression and intervention. However, the further modifications to the original ATN classification should be tested to improve the deficiencies in clinical application, and prospective studies with larger sample sizes investigating the pattern of MM progression based on the ATN classification system will be necessary to further confirm the validity of this classification system.

## Supplementary Information

**Additional file 1:** **Sfigure.** Main characteristics of lesion types and sub-types.

## Data Availability

The datasets analyzed during the current study are available from the corresponding author on reasonable request.

## Abbreviations

MM: Myopic maculopathy
PM: Pathologic myopia
OCT: Optical coherence tomography
A: Atrophy
T: Traction
N: Neovascularization
κ: Kappa coefficient
CI: Confidence interval
HM: High myopia
MTM: Myopic traction maculopathy
DSM: Dome shaped macula
PPV: Pars plana vitrectomy
MB: Macular buckle
ILM: Internal limiting membrane
ELM: External limiting membrane
RPE: Retinal pigment epithelium
CNV: Choroidal neovascularization
CT: Choroidal thickness
PDCA: Peripapillary diffuse choroidal atrophy
LCs: Lacquer cracks
