# Supplementary material for: Intra- and interobserver concordance of a new classification system for myopic maculopathy
Source: BMC Ophthalmol. 2021 Apr 23;21:187. doi: 10.1186/s12886-021-01940-4 (PMC8063469; doi:10.1186/s12886-021-01940-4)
Supplement: Supplementary file 1 [file 12886_2021_1940_MOESM1_ESM.docx]

## Slide 1

Type A component: Mainly based on the fundus photographs
Atrophy(A): a:No myopic retinal lesions (A0); b:Tessellated fundus (A1); c:Diffuse chorioretinal atrophy (A2); d:Patchy chorioretinal atrophy (A3); e:Patchy atrophy-related macular atrophy (A4); f:CNV-related macular atrophy (A4)

a

b

d

c

e

f

## Slide 2

Type T component: mainly based on the OCT
Traction (T): a:No macular schisis (T0); b:Inner foveoschisis (T1); c:Outer foveoschisis (T1); d:Inner and outer foveoschisis (T2); e:Foveal detachment (T3); f:Full-thickness macular hole (T4); g:Macular hole retinal detachment (T5) 

a

b

c

d

f

g

e

## Slide 3

Type N component: based on the fundus and OCT
Neovascularization (N):a:No myopic CNV (N0); b:Macular lacquer cracks (N1); c:Active CNV (N2a); d:Scar/Fuch’s spot (N2s)

b

c

d

a
